# Novel Tools for Conservation Genomics: Comparing Two High-Throughput Approaches for SNP Discovery in the Transcriptome of the European Hake

**DOI:** 10.1371/journal.pone.0028008

**Published:** 2011-11-22

**Authors:** Ilaria Milano, Massimiliano Babbucci, Frank Panitz, Rob Ogden, Rasmus O. Nielsen, Martin I. Taylor, Sarah J. Helyar, Gary R. Carvalho, Montserrat Espiñeira, Miroslava Atanassova, Fausto Tinti, Gregory E. Maes, Tomaso Patarnello, FishPopTrace Consortium, Luca Bargelloni

**Affiliations:** 1 Department of Experimental and Evolutionary Biology, University of Bologna, Bologna, Italy; 2 Department of Public Health, Comparative Pathology, and Veterinary Hygiene, University of Padova, Legnaro, Italy; 3 Department of Molecular Biology and Genetics, Faculty of Science and Technology, Aarhus University, Tjele, Denmark; 4 TRACE Wildlife Forensics Network, Royal Zoological Society of Scotland, Edinburgh, United Kingdom; 5 Molecular Ecology and Fisheries Genetics Laboratory (MEFGL), School of Biological Sciences, Environment Centre Wales, University of Bangor, Bangor, Gwynedd, United Kingdom; 6 ANFACO-CECOPECSA, Vigo, Spain; 7 Laboratory of Animal Diversity and Systematics, Katholieke Universiteit Leuven, Leuven, Belgium; Auburn University, United States of America

## Abstract

The growing accessibility to genomic resources using next-generation sequencing (NGS) technologies has revolutionized the application of molecular genetic tools to ecology and evolutionary studies in non-model organisms. Here we present the case study of the European hake (*Merluccius merluccius*), one of the most important demersal resources of European fisheries. Two sequencing platforms, the Roche 454 FLX (454) and the Illumina Genome Analyzer (GAII), were used for Single Nucleotide Polymorphisms (SNPs) discovery in the hake muscle transcriptome. *De novo* transcriptome assembly into unique contigs, annotation, and *in silico* SNP detection were carried out in parallel for 454 and GAII sequence data. High-throughput genotyping using the Illumina GoldenGate assay was performed for validating 1,536 putative SNPs. Validation results were analysed to compare the performances of 454 and GAII methods and to evaluate the role of several variables (*e.g.* sequencing depth, intron-exon structure, sequence quality and annotation). Despite well-known differences in sequence length and throughput, the two approaches showed similar assay conversion rates (approximately 43%) and percentages of polymorphic loci (67.5% and 63.3% for GAII and 454, respectively). Both NGS platforms therefore demonstrated to be suitable for large scale identification of SNPs in transcribed regions of non-model species, although the lack of a reference genome profoundly affects the genotyping success rate. The overall efficiency, however, can be improved using strict quality and filtering criteria for SNP selection (sequence quality, intron-exon structure, target region score).

## Introduction

The European hake (*Merluccius merluccius*, Linnaeus, 1758; Merlucciidae, Actinopterygii) is a widely distributed species inhabiting the North-Eastern Atlantic Ocean and the Mediterranean Sea, whose respective stocks are managed by the International Council for the Exploration of the Seas (ICES) and the General Fisheries Commission for the Mediterranean (GFCM). It represents one of the most important demersal target species of Western European and Mediterranean commercial fisheries [1], [2]. According to the FAO report on 2008 fishery statistics [3], European hake ranks first within Mediterranean and among the ten top demersal fish species in North-East Atlantic in terms of catches, and at present stocks are under heavy exploitation and rebuilding action plans [4], [5]. Despite its relevance as a fishery resource and growing concerns on fishery stock sustainability, population structure and management units of European hake are only roughly defined and poorly supported by fishery-independent data, such as life-history traits and population genetics. At present, three large stock units of European hake have been recognized in a management framework, two in the North-Eastern Atlantic (*i.e.* the Northern and Southern stocks which extend northward and southward to the Cape Breton submarine canyon in the Bay of Biscay, respectively) and one in the whole Mediterranean, from the Gibraltar Strait to the Levantine Sea. Population genetic structure assessed at six potentially neutral microsatellite loci supported a distinction between Atlantic and Mediterranean stocks [6], [7], while the high connectivity and temporal variation among Atlantic populations [6]–[10] questioned the separation between Northern and Southern Atlantic stocks [11], [12]. However, the subtle genetic differentiation among population samples at the neutral loci revealed a relatively weak population structure and prevented the robust assignment of individual fish to the stock of origin [7]. On the other hand, genetic loci potentially under selection might be more efficient in detecting locally adapted populations and associated potential management units for conservation in marine fish [13], [14]. In European hake populations a significant correlation between the allele frequency variation at the glyceraldehyde-3-phosphate dehydrogenase locus and the latitudinal gradient in surface salinity has been previously detected [15]. Identification of other genetic loci potentially under selection might provide promising tools to resolve population structure and traceability of this species at a smaller spatial scale.

Recently, major technological advances have opened innovative opportunities in the application of molecular genetic tools to study evolutionary processes in natural populations of marine species [14], [16]. The emergent field of population genomics [17] has shown the potential of exploring genetic variation at a genome-wide scale and offered the opportunity to gain insights into the evolutionary mechanisms of adaptive divergence in the wild [18]–[20]. The growing interest in developing genomic resources is due mainly to the rapid development of next-generation sequencing (NGS) technologies (see review by Metzker [21]), allowing the production of massive volumes of data at relatively modest and decreasing costs compared to the traditional Sanger sequencing method. The opportunity to obtain extremely large collections of expressed sequence tags (ESTs) at reduced cost, potentially provides an unprecedented trove of genetic markers located in functionally relevant regions of the genome [22]–[26]. Although EST data mining has mainly been used so far to identify microsatellite loci, the application of single nucleotide polymorphisms (SNPs) is rapidly catching up [27], because they are the most abundant and widespread genetic variants in the eukaryote genome, with great potential in ecological and evolutionary studies [28], [29]. Compared to microsatellites, SNPs show lower genotyping error rates, higher data quality and genomic coverage and low probability of homoplasy [28], with the further advantage of easier “portability” of genotypic data across laboratories. A major benefit of utilizing SNP markers associated with transcribed regions of the genome concerns the prospect of identifying outlier genetic markers, showing significantly increased or decreased differentiation among populations compared to neutral expectations [17], [30]. Outlier loci are presumed to be either directly linked or in tight linkage disequilibrium with loci subject to natural selection [19], [31]. Scaling up the number of available EST-linked SNPs to hundreds of thousands of markers extends the genome-coverage, thereby increasing the probability of identifying loci under selection, and associated insights into population structuring and its determinants [32].

The growing accessibility to high-throughput sequencing technologies and the concurrent development of innovative bioinformatics tools has enabled the application of wide-scale SNP discovery based on transcriptome sequencing even in species for which genomic resources are still limited or absent [25], [26], [33]–[37]. Recently, several studies concerning fish species relevant to fishery and aquaculture, such as rainbow trout [38], lake sturgeon [39], lake whitefish [40], sockeye salmon [41], catfish [42] and turbot [43] have successfully used this approach. However, experimental evidence on large scale validation of *in silico*-identified SNPs is still limited and restricted to EST data sets obtained with traditional Sanger sequencing [44], [45].

The present study reports the muscle transcriptome characterization and the discovery and validation of a large set of SNP loci for European hake, based on NGS technologies. Existing NGS technologies provide a variety of approaches for transcriptome characterization, although the two most popular platforms are the Roche 454 FLX [46] and the Illumina Genome Analyzer [47], [48]. The Roche 454 FLX (hereafter 454) is capable of generating one hundred million nucleotides per run, producing sequences of length approaching 400 base pairs (bp) with an average substitution error rate that is relatively low with respect to other sequencing technologies. The alternative leading sequencing system, the Illumina Genome Analyzer (hereafter GAII) produces shorter reads, currently up to 150 bp, with higher average substitution error rates but much higher throughput and lower costs [48]. As longer reads facilitate *de novo* assembly most published studies on transcriptome sequencing of non-model organism have to date used the Roche 454 pyrosequencing platform; however current advances in bioinformatics have allowed the assembly of shorter reads without reference genome or transcriptome [41], [49]. Using the European hake as a case study for non-model species, these two approaches (454 and GAII) were applied in parallel to high-throughput SNP discovery in expressed sequences. Large scale (1,536 SNPs) validation of discovered markers was carried out to properly evaluate and compare their performance. The ultimate goal was to test whether an optimal and cost effective strategy exists to develop a broad set of SNP markers linked to functional loci to be used for improved genetic management of hake fisheries.

## Materials and Methods

### Samples for cDNA libraries

**Figure 1:**
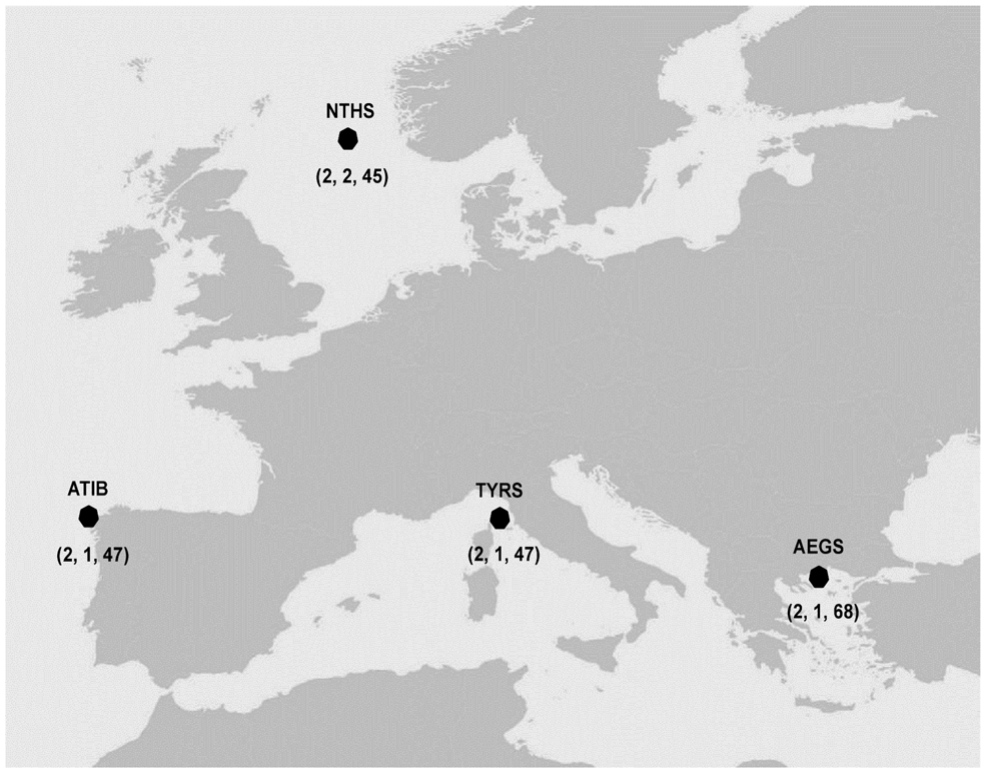
Geographic location of the four sampling sites. In brackets the number of specimens in the discovery panel (454 and GAII) and the number of individuals that have been subsequently genotyped in the validation step. NTHS: North Sea (59°19′N, 1°39′E); ATIB: Iberian Atlantic coast (43°20′N, 8°56′W); TYRS: Tyrrhenian Sea (42°32′N, 10°9′E); AEGS: Aegean Sea (40°19′N, 24°33′E).

Muscle tissue samples for transcriptome sequencing were collected from four geographic regions (Figure 1) considered representative for the species range: two locations in the Mediterranean Sea (Aegean Sea and North Tyrrhenian, hereafter respectively AEGS and TYRS), and two in the Atlantic Ocean (North Sea and Iberian Atlantic coast, hereafter NTHS and ATIB, respectively). Animals used in this research were obtained from commercial fishery catches, therefore approval from any ethics committee or institutional review board was not necessary. Muscle tissues were stored in RNA later (Qiagen) at −80°C before RNA extraction. Two distinct non-normalized cDNA libraries were constructed and sequenced using 454 and GAII sequencing systems, following the methods described in detail below. Non-normalized library were used since, according to Hale *et al.* [39], cDNA libraries normalization has little impact on discovery of rare transcripts when NGS platform are used, due to the depth of sequencing coverage.

### RNA extraction, cDNA library construction and 454 GS FLX sequencing

Total mRNA was obtained from eight individuals (two for each sampling location, Figure 1) using the RNeasy Lipid Tissue Mini Kit (Qiagen). mRNA was isolated using the Oligotex mRNA Mini Kit (Qiagen) and cDNA was synthesized using the SuperScript Double-stranded cDNA Synthesis Kit (Invitrogen). Due to the low amount of available cDNA compared to that specified in standard protocol for the preparation of a GS FLX sequencing library using Roche multiplex identifiers (MIDs), it was decided to use a customized barcoding protocol, modified from Binladen *et al.* [50], that allows smaller amounts of cDNA to be used for library preparation: a multiplex sequencing library was prepared by labeling each individual sample with two tags by ligation of specific 10-mer barcoding oligonucleotides to allow post-sequencing identification of sequences from the different samples. High-throughput sequencing was performed on a Roche 454 GS FLX (454) sequencer according to the manufacturer's protocol.

### RNA extraction, cDNA library construction and Illumina Genome Analyzer II sequencing

Total RNA was purified from muscle tissue from five individual samples, two for NTHS, and one from each of the three other sampling sites (Figure 1) using the mRNeasy isolation kit (Qiagen) following the manufacture's guidelines. A total of 10 µg total RNA from each sample were used for library construction following the protocol described by Marioni *et al.* [51]. Poly-A containing mRNA was purified using poly-T oligonucleotide attached magnetic beads and fragmented using divalent cations under elevated temperature. After copying the RNA fragments into first strand cDNA using reverse transcriptase and random hexamer primers, second strand cDNA synthesis was performed using DNA Polymerase I and RNaseH. The short cDNA fragments were then prepared for sequencing using the Genomic DNA Sequencing Sample Prep Kit (Illumina). Briefly, the cDNA fragments were “end-repaired” using T4 DNA polymerase and Klenow DNA polymerase before adding a single A base to the cDNA fragments by using 3′-to-5′ exo-nuclease. Following ligation of Illumina adaptors, fragments of approximately 200 bp in size were gel purified and enriched by PCR amplification for 15 cycles. Library concentrations were measured using a Qubit fluorometer (Invitrogen) and size and purity were assessed using an Agilent 2100 Bioanalyzer (Agilent DNA 1000 Kit). Following dilution in buffer EB (Qiagen) to 10 nM, the libraries were denaturated with 2 M NaOH to a final DNA concentration of 1.0 nM, diluted to 4 pM with pre-chilled Hybridization buffer (Illumina) before loading in individual lanes into a 1.0 mm flowcell together with a single lane of a 2 pM PhiX control library (Illumina). Sequencing (76 cycles) was conducted on an Illumina Genome Analyzer (version II) using the Genomic DNA sequencing primer in combination with clustering and sequencing kits supplied by Illumina. Analysis of the images taken during sequencing was performed using the Genome Analyzer Pipeline Software (version 1.4.0, Illumina) generating the raw fastq files.

### 454 sequence processing and SNP detection

The sequences were de-multiplexed based on the specific barcoding tags and binned per individual sample. In order to obtain an optimal *de novo* assembly of sequences, repeats and repetitive or low complexity sequences were identified and masked in the reads by RepeatMasker (version open-3.2.7 with RM database version 20090120; Smit AFA, Hubley R & Green P: RepeatMasker at http://repeatmasker.org) using the Zebrafish (*Danio rerio*) repeat library and then cleaned for short sequences using SeqClean (http://compbio.dfci.harvard.edu/tgi/software/; options: -N; -L50). Sequence clustering was performed using CLC GenomicsWorkbench (parameters: match mode = ignore; similarity = 0.99; length fraction = 0.5; insertion cost = 3; deletion cost = 3; mismatch cost = 2) and after parsing the resulting large ace file respective sequences were then assembled ‘per contig’ using the assembly program CAP3 [52] (options: -o50; -p94) in order to generate the consensus sequences to be used as reference for read mapping in the subsequent SNP detection step. The GigaBayes program, a new implementation and expansion of the PolyBayes SNP detection algorithm by Marth *et al.* [53], was used for SNP discovery (parameters: ploidy = diploid; CRL = 4; CAL = 2; O = 3; D = 0.003), applying a minimum contig depth of four reads covering the polymorphic site with at least two reads for each allele; no insertion or deletion variants (InDels) were considered.

### GAII sequence processing and SNP detection

After renaming and trimming of the first base, GAII short reads were assembled *de novo* using Abyss [54], [55]. Different settings were tested and the final assembly was run using a k-mer value of 65. Contig trimming and exclusion of sequences shorter than 100 bp was performed by Seqclean (http://compbio.dfci.harvard.edu/tgi/software/; l = 100). MosaikBuild was used to build a database of reads (options: -st illumina, -tp 1, -ts 1) and MosaikAligner was applied (options: -mmp 0.05, -mm 12, unique, -act 35) to map the reads (database) against the Abyss contigs. After running MosaikSort the MosaikAssembler was used to generate the (.gig) input files for the SNP detection by GigaBayes (options: gff, diploid, multiple, CRL 10, CAL 4, D 0.003).

### Contig functional annotation and Gene Ontology analyses

In order to characterize 454 and GAII contigs several approaches were explored, using the Basic Local Alignment Search Tool (BLAST) against various protein and nucleotide databases. The Blastn option was used against the NCBI nucleotide database (cut-off e-value of <1.0 E^−5^), against all annotated transcripts in the draft genomes of *Danio rerio*, *Gasterosteus aculeatus*, *Oryzias latipes*, *Takifugu rubripes*, *Tetraodon nigroviridis*, and *Homo sapiens* available at the Ensembl Genome Browser, and against all unique transcripts for *D. rerio*, *H. sapiens*, *O. latipes*, *T. rubripes*, *Salmo salar*, *Oncorhynchus mykiss* stored in the NCBI UniGene databases. The Blastx option was used (cut-off e-value of <1.0 E^−3^) to search against the entire UniProtKB/SwissProt and UniProtKB/TrEMBL protein databases as well as against the annotated proteins from the transcriptomes of *D. rerio*, *G. aculeatus*, *O. latipes*, *T. rubripes*, *T. nigroviridis*, and *H. sapiens* available through the Ensembl Genome Browser.

The Gene Ontology (GO) terms (“Cellular Component”, “Biological Process” and “Molecular Function”) were recovered using the Blastx search tool implemented in the software Blast2GO [56] against the NCBI non-redundant protein database.

A pipeline was developed to characterize the SNP mutations at the amino acid level. To obtain the putative reading frame all SNP-containing contigs were compared against six peptide sequence databases (Ensembl genome assembly for *G. aculeatus*, *T. nigroviridis*, *O. latipes*, *T. rubripes*, *D. rerio* and Swissprot database) using the Blastx algorithm (cut-off e-value of <1.0 E^−3^). The best match was selected and the aligned sequence portions of the query were saved as fasta files and then formatted as a Blast database. A fasta file containing 120 bp SNP-flanking sequences was prepared (two sequences for both alleles of each SNP) and a Blastx analysis was performed against the previously formatted database (cut-off e-value of <1.0 E^−10^). The aligned sequence portion of the two alleles for each SNP was compared for the presence of a synonymous or not-synonymous mutation.

To evaluate whether SNP-containing contigs were significantly enriched for specific GO terms compared to all annotated hake contigs, the Gossip package [57], which is integrated in the Blast2Go software, was used. Statistical assessment of annotation differences between the two sets of sequences (SNP-containing contigs vs all hake contigs) was carried out using Fisher's Exact Test with False Discovery Rate (FDR) correction for multiple testing.

### Candidate SNP selection

After SNP detection, *in silico* evaluation of candidate SNPs was carried out to select a panel of 1,536 candidate SNPs for high-throughput genotyping validation. Selection criteria were based on the score assigned by the Illumina GoldenGate Assay Design Tool (ADT), the analysis of putative intron-exon boundaries within each contig, and visual inspection of flanking region sequence quality. SNP scores obtained with the ADT take into consideration template GC content, melting temperature, uniqueness, and tendency to form hairpin loops. All SNPs with ADT scores below 0.4 were discarded, while SNPs with an ADT score higher than 0.7 were preferentially selected.

Intron-exon boundary prediction was performed using fish genome and transcriptome sequence resources following two parallel approaches. In the first, SNP-containing contigs were compared against five high-quality draft fish genomes (Ensembl genome assemblies for *G. aculeatus*, *T. nigroviridis*, *O. latipes*, *T. rubripes*, and *D. rerio*) using Blastn (cut-off e-value of <1.0 E^−5^). After parsing Blast results, the best match was listed including information on alignment length, as well as the start and end of the aligned region. In the case of a positive match, the position of the candidate SNP was evaluated in the framework of the aligned region. If the 60 bp up- and downstream of the SNP position were present in the alignment, the candidate SNP was considered embedded in a single exon, otherwise an intron was assumed to be present in the 121 bp target region for SNP assay design. The same process was repeated against five different databases (see above) and each SNP was assigned a code, either “1” (when in at least one comparison the candidate SNP and its flanking regions were located on a single exon), “0” (an intron was predicted to disrupt the candidate region), or “no” (no significant match against any of the five reference fish genomes). The second approach was designed in order to further increase the likelihood of a positive match and the reliability of intron-exon boundary prediction. SNP-containing contigs were used as a query in a Blast search (Blastn option, cut-off e-value of <1.0 E^−5^) against the transcriptome of each of the five model species as above. In the case of a positive hit, the matching transcript for each fish model species was downloaded from the Ensembl database, and the nucleotide position in the downloaded sequence corresponding to the candidate SNP in the original hake contig was identified based on the start-end positions of the Blast alignment between the hake contig and the fish model transcript. Then the putative homolog transcript was compared to its own genome sequence using Blast. Based on the inferred SNP position on the fish model transcript, SNPs were assigned a code as above (“1” for SNP candidate region located on a single exon or “0” if SNP region was assumed to be disrupted by an intron). In the case of no matches between the original hake contig and any of the five fish transcriptomes, the SNP was scored “no”. A flowchart for the intron-exon pipeline is depicted in Figure S3.

A final evaluation step of putative SNPs was performed by direct visual inspection of contigs using the assembly viewer software Eagleview [58] and Cluster Viewer (clview; http://compbio.dfci.harvard.edu/tgi/software/), with the aim of ranking candidates within each contig by integrating information on the overall contig assembly quality, depth and length, the quality of flanking regions (number of ambiguous sites), distance and clustering of polymorphic sites. SNPs with highest rank values were selected within each contig.

### SNP validation by high-throughput genotyping

A total of 1,536 candidate SNPs were selected to be validated by high-throughput genotyping. Genomic DNA was extracted from fin clip tissues of 207 individuals sampled from the same four locations of origin of specimens used to derive the libraries (AEGS, TYRS, ATIB, NTHS, see Figure 1). A NanoDrop spectrophotomer was used to ascertain that DNA quality and quantity met the requirements for the genotyping assay. Genotyping was performed using the Illumina GoldenGate Assay platform [59] and the resulting data were visualized and analyzed with the GenomeStudio Data Analysis Software package (1.0.2.20706, Illumina Inc.). Samples with a call rate lower than 0.8 and loci showing poor clustering were excluded. Accepted SNPs were manually re-clustered, to correct errors in allele calling due to inappropriate cluster identification.

### Statistical analysis

Different variables were defined to analyze results of SNP discovery and genotyping. The first two are categorical variables that refer to the outcome of individual SNP assays. *SNP_assay_conversion* assumes value 0 (failed) if Illumina SNP assay did not yield a reliable genotype for the examined individuals, either because no clear clustering was observed or due to lack of signal, value 1 (successful) if consistent clustering was obtained, irrespective of the observed genotype(s). *SNP_genotype* has value 0 (monomorphic) if all scored individuals are homozygous for the same allele, value 1 (polymorphic) if two alleles are observed. *SNP_score* reports ADT score for individual SNPs. *I_E_test* and *I_E_species_match* refer to the outcome of the intron-exon boundary analysis pipeline, *I_E_test* = “no” means no significant match could be obtained, value 1 signifies that at least one significant match was reported, with the SNP candidate region putatively contained in a single exonic region, while *I_E_species_match* counts the number of fish model species showing a significant Blast match. *Depth* is a quantitative variable reporting the number of sequence reads supporting individual SNPs, *Individuals* and *Geosites* counts respectively the number of individuals and geographical sites from the original discovery panel that contributed with at least one sequence read to a specific SNP. *MSAF* (minor sequencing allele frequency) represents the frequency of the minor allele detected at the SNP discovery stage. *Rank* is an ordinal variable referring to an order of choice of SNPs within each contig, arbitrarily assigned after a visual inspection (see above). *Q* reports the number of mismatches in the flanking regions of SNP positions based on comparison of sequence reads within a contig. SNP discovery results obtained using 454 sequencing technology were compared with those produced through GAII sequencing using either parametric T-tests for two independent samples (for quantitative, normally distributed variables: *Contig length*, *Depth*) or non-parametric tests, a χ^2^ test for categorical variables (*SNP_assay_conversion*, *SNP_genotype*, *I_E_test*) and a Mann-Whitney U test for ordinal variables (*I_E_species_match*, *Geosites*) or quantitative variables not following a normal distribution (*SNP_score*, *MSAF*). All tests were carried out in SPSS ver. 12.0 with Monte Carlo simulation (1,000,000 permutations) to estimate confidence intervals for Mann-Whitney tests.

Binomial logistic regression, implemented in SPSS ver. 12.0, was used to evaluate several predictor variables on two dependent dichotomous variables: *SNP_assay_conversion* and *SNP_genotype*. For both data sets (GAII and 454), positive conversion/clustering of individual SNP assay was analyzed first, assigning single SNPs into two classes (successful-failed). Second, DNA polymorphism was evaluated by filtering out failed SNPs and dividing positive loci respectively into two groups based on observed genotypes (polymorphic-monomorphic, *SNP_genotype*). For 454 data, eight ordinal/quantitative variables (*SNP_score*, *I_E_species_match*, *Depth*, *MSAF*, *Individuals*, *Geosites*, *Q*, *Rank*) and one categorical variable (*I_E_test*) were considered. For GAII data, six ordinal/quantitative variables (*SNP_score*, *I_E_species_match*, *Depth*, *MSAF*, *Individuals*, *Geosites*) and one categorical variable (*I_E_test*) were examined. Predictor variables were either all included in the predicting model (option “enter” in SPSS) or best predictors were selected using a stepwise deletion approach (option “backward”). In the latter approach, the Wald χ^2^ statistic was used to estimate the contribution of each predictor.

The Receiver Operating Characteristic (ROC) curve, a widely used method for evaluating the discriminating power of a diagnostic test, was used to assess the significance of specific variables. ROC analysis was implemented in MedCalc ver. 11.5.1.0.

## Results and Discussion

### Sequencing, *de novo* assembly, and annotation

Approximately 100 Mega base pairs (Mbp) were obtained using 454 sequencing technology, whereas nearly 4,000 Mbp were produced using the GAII sequencer (Table 1). About 6% (30,025) of raw 454 reads, in which no barcoding tag could be reliably recognized, were excluded from further analysis, as individual sample identity of the reads was considered essential to the validation process. The remaining 476,747 reads, which showed the 5′ barcoding tag, were assigned to four groups according to geographic origin (Figure 1, 102,854 for AEGS, 135,494 for TYRS, 97,207 for NTHS, and 141,192 for ATIB). GAII sequencing yielded 8,789,024, 10,327,499, and 10,029,014 reads from single individuals from AEGS, TYRS, and ATIB respectively, while 21,539,868 sequences were obtained from two distinct individuals from NTHS. All 454 and GAII sequence data have been submitted to the EBI Sequence Read Archive (SRA) under the study accession number ERP000950 (http://www.ebi.ac.uk/ena/data/view/ERP000950).

**Table 1:** Summary statistics of sequence assembly.

	454	GAII
**Total number of sequences**	506,772	50,685,405
**Average length (min-max)**	206 (6–457) bp	74 bp
**Sequences suitable for assembly**	462,489	50,685,405
**Number of contigs**	5,702	3,756
**Average contig length (min-max)**	331 (100–5,103) bp	190 (100–3,063) bp
**Annotated contigs (%)**	4,221 (74.03)	2,644 (70.39)

After pre-processing steps (adaptor clipping and read quality filtering), 462,489 454 reads were assembled *de novo* into 5,702 separate contigs of at least 100 bp length (from 4,710 initial clusters). GAII sequence assembly resulted in 9,258 contigs, of which 60% were discarded, having a length shorter than 100 bp. The remaining 3,756 contigs were further processed for SNP discovery. Results on contig length are summarized in Table 1. As expected, mean contig length was significantly higher for 454 than GAII (T-test p<0.0001), whereas the opposite was observed for sequence coverage (T-test p<0.0001). The observed average length of unique transcripts after assembly of 454 and GAII sequence reads are comparable with values reported in a recent study that applied a simulation approach (based on experimental data) to evaluate the performance of transcriptome sequencing using Sanger, Roche 454, and GAII technologies on either non-normalized or normalized cDNA libraries [47]. For 454 GFLX sequencing on non-normalized libraries (100 Mbp output) average contig length is expected to be 556.6 bp and 217.3 bp for GAII sequencing (4,000 Mbp output, non-normalized libraries) [47], similar to what was observed in the present study, whereas the number of unique contigs obtained for hake muscle transcriptome is considerably smaller than expected (respectively 37,853 contigs for 454 and 147,261 for GAII under the same conditions as above). Differences in the experimental setting in the present work (*e.g.* use of a single tissue library and different genome size of the target species) are likely contributing factors to the observed differences.

A total of 4,221 454-contigs (74.02%) showed a significant Blast match against at least one species sequence database among those searched. Annotation by similarity was possible for 2,644 GAII contigs (70.39%), significantly less (χ^2^ = 14.83, p<0.001) than 454 contigs. A potential explanation for this observation lies in the shorter average length of GAII contigs, which likely reduces the overall probability of obtaining positive Blast hits. In any case, the percentage of annotated contigs is higher for both 454 and GAII data when compared to other studies reporting transcriptome sequencing in teleost fish (40–63% of total annotated contigs, [45], [60]). There are several variables that might influence the observed fraction of contigs with a positive Blast match, including phylogenetic distance from species with high quality draft genome sequence, contig length, tissue type(s), developmental stages, and the method used for library construction. The use of a non-normalized cDNA library of adult skeletal muscle may have led to a biased representation of the hake transcriptome, favouring highly expressed genes encoding either housekeeping or structural (muscle contractile fibres) proteins. Housekeeping and structural proteins are known to be expressed at higher levels and to show a higher degree of sequence conservation compared to other proteins [61] (*e.g.* components of the immune system), therefore increasing the chance of finding a positive Blast match. A total of 120 454-contigs (2.1%) and 67 GAII-contigs (1.78%) were identified as mitochondrial sequences. It was possible to associate one or more GO terms to 1,606 454-contigs (28.16%) and 884 GAII contigs (23.53%). Results from level 2 GO assignments within the three categories are summarized in Figures S1 and S2.

### *In silico* SNP detection

**Figure 2:**
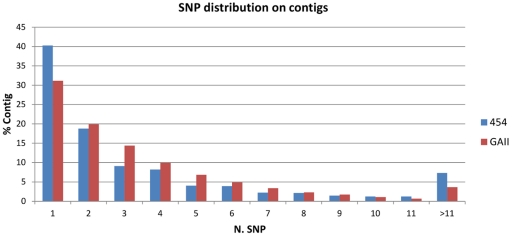
Distribution of SNPs across 454 and GAII contigs. On the x-axis, number of SNPs per contig; on the y-axis, the percentage of contigs showing a specific number of SNPs.

A total of 4,034 candidate SNPs were identified *in silico* in 889 454-sequenced contigs (15%), with an average of 0.73 SNPs per 100 bp surveyed in the assembled transcriptome, or 41.1 SNPs per generated Mbp. Approximately 60% of 454-contigs contained one or two SNPs (Figure 2). The total number of SNPs (8,606) found in 2,384 GAII contigs was more than two-fold higher, with a comparable distribution across contigs (Figure 2). A mean of 1.7 SNPs was found every 100 analysed bp, but with a lower output (2.3) per sequenced Mbp. As already observed, sequencing depth at candidate SNP positions was significantly higher (T-test p<0.0001) in GAII sequence data (Table 2), which might also explain the near two-fold increase in frequency of SNPs per bp reported above. On the other hand, 3,621 SNPs (89.7%) from 454-generated contigs had at least 60 bp of flanking sequence on either side of the SNP, the minimal requirement for the Illumina GoldenGate genotyping assay, while nearly half of GAII-generated SNPs were discarded due to flanking regions of insufficient size, an observation directly related to the highly significant difference (T-test p<0.0001) in the average length of SNP-containing contigs (Table 2). A comparison of 454- and GAII-SNPs revealed that 440 loci were common to both data sets. All suitable SNPs (*i.e.* having sufficient flanking regions) were analysed with the Illumina ADT software, and SNPs with ADT score >0.4 (3,437) were further evaluated. After filtering for the intron-exon boundary prediction results, 2,173 SNPs in the 454 data, presumed to be located in single exons (851 SNPs) or without a Blast match (1,322 SNPs), were selected to be visually inspected. For GAII SNPs, 3,857 loci out of 4,637 with ADT score >0.4 passed the filtering step based on the intron-exon boundary prediction (468 located in single exons and 3,389 with no significant match) (Table 2).

**Table 2:** Summary statistics of SNP discovery and selection.

	454	GAII
***In silico*** ** candidate SNPs**	4,034	8,606
**Contigs with candidate SNPs**	889	2,384
**Average contig length (min-max)**	617.9 (101–5103) bp	212.3 (100–3063) bp
**Average depth in SNP position (min-max)**	89 (4–3,678)	674 (8–33,079)
**SNPs suitable for Illumina assay design**	3,621	4,684
**Average ADT score**	0.76	0.82
**SNPs with ADT score >0.4 (%)**	3,437 (94.92%)	4,637 (99%)
**SNPs with I/E** * **“no match” (%)**	1,322 (38.46%)	3,389 (73.09%)
**SNPs with I/E** * **“single exon” (%)**	851 (24.76%)	468 (10.09%)

*Intron/exon boundary pipeline result.

**Figure 3:**
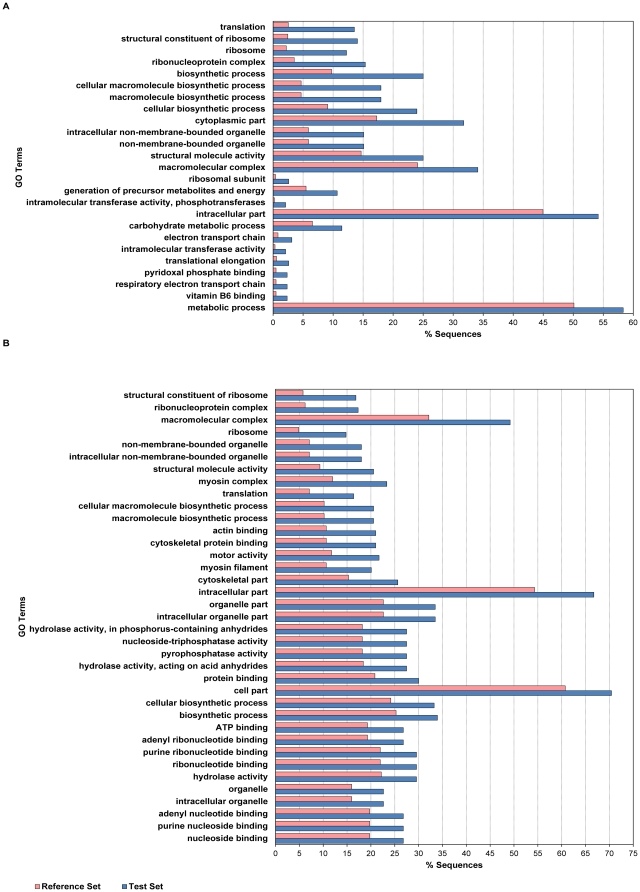
Enrichment of SNP-containing contigs in GO terms. Differential distribution of GO terms in SNP-containing contigs (test set) compared to all contigs (reference set) in 454 data (A) and GAII data (B).

Annotation by similarity of SNP-containing contigs further confirmed that 454-contigs show a higher percentage of putatively annotated sequences (86% vs 71%, χ^2^ = 72.4, p<0.0001). Blast searches also identified 218 candidate SNPs (positioned in 62 different contigs) that are presumably located on the hake mitochondrial genome. GO term analysis showed a significant enrichment of specific GO terms when comparing the annotations of SNP-containing contigs against all unique transcripts obtained for the hake muscle transcriptome (Figure 3 A, B, Tables S1 and S2 in Supporting Information). Protein synthesis, in particular ribosome assembly/function, and energetic metabolism (*e.g.* carbohydrate metabolism, electron chain transport, ATP synthesis) are significantly over-represented across both 454- and GAII-SNPs, while cytoskeletal components (*e.g.* myosin filaments, microtubules) are enriched in GAII-SNPs. As mentioned before, the use of non-normalized cDNA libraries likely favored a higher representation of abundantly expressed genes. In the skeletal muscle, a high level of protein synthesis is required to fulfill the need for abundant contractile fibres, therefore over-representation of ribosomal/translation components as well as cytoskeletal proteins is expected. Such over-representation likely translates into greater sequence coverage and ultimately in a larger proportion of SNPs being identified in specific functional groups of genes.

### 454 and GAII SNP validation

A set of 1,536 SNPs (Table 3) was selected mainly on the basis of ADT score and intron/exon analysis for validation on the Illumina Golden Gate platform, using a custom-designed SNP chip. Mitochondrial SNPs (35) were also validated, but were excluded from further analysis as they are not entirely comparable (*i.e.* mitochondrial DNA is a multiple copy, haploid, and intron-less genome). In total, 817 454-SNPs distributed on 516 contigs, 557 GAII-SNPs distributed on 463 contigs, 127 common (454 and GAII) nuclear SNPs, and 35 mitochondrial loci were included in the panel of 1,536 candidate loci to be validated by high-throughput genotyping. The common set of 127 nuclear SNPs was genotyped only once, but all these loci have been independently identified twice and descriptive variables could be estimated in both data sets (454 and GAII). For this reason, they have been considered as independent observations and analysed separately.

**Table 3:** Summary statistics for SNP validation.

	454	GAII
**Total number of SNPs tested**	966 (829)	707 (570)
**Nuclear SNPs tested**	944 (817)	684 (557)
**SNPs with successful genotype calling** *	409 (334)	296 (221)
**Polymorphic**	259 (195)	200 (136)
**SNP with known reading frame**	130 (97)	73 (45)
**Synonymous**	110 (83)	60 (37)
**Non-synonymous**	20 (14)	13 (8)
**Monomorphic**	150 (139)	96 (85)
**Failed SNPs** *	535 (483)	388 (336)

In brackets the number of SNPs after excluding the set of common loci.

*Data referring to nuclear SNPs.

Excluding mitochondrial loci, 1,501 unique nuclear SNPs were scored, with nearly identical percentages of successful assay conversion for the two sets of data (409/944, 43.32% (454) and 296/684, 43.27% (GAII), χ^2^ = 0, p = 1). This leads to the question of how do SNP conversion rates compare to those observed in other studies that reported on high-throughput SNP discovery and validation in non-model species. This remains largely unexplored, and the few available data are not homogeneous. Most studies report *in silico* SNP detection in next-generation sequencing data from non-model species with limited, if any, experimental validation of discovered polymorphisms (*e.g.* [29], [33], [35], [42]). In a recent study on SNP discovery and validation in salmonid species [41], pooled and single tissue cDNA libraries were sequenced with SOLiD technology and short sequence reads aligned to contigs obtained from reference EST databases. This approach yielded a similar number of average SNPs per contig (1.6–4.4) to that observed here (3.6–4.5, Table 2). PCR-based validation was carried out on 96 SNPs, with an assay conversion success rate of 53% (51/96). The conversion rate of validation assays (Illumina Golden Gate) was found to be much higher in two larger studies carried out in non model bird species, the common turkey [62] and the mallard duck [63], for which rates of 88.5% (340/384) and 94.7% (364/384) were observed, respectively. Both studies, however, used Illumina GA technology to sequence reduced representation (genomic) libraries (RRL) of the target species genome. Drastic sequence quality filtering was enforced especially in the turkey study, discarding up to 75% of sequence reads. Finally, assembled turkey contigs were verified through mapping against the high quality draft genome of the closely related chicken species. This step appears crucial as shown by the comparison of conversion rates between genome-mapped turkey SNPs (92.1%, 316/343) and unmapped ones (58%, 24/41) [62]. Likewise, mallard duck genomic contigs were directly aligned against the domestic duck genome, which provided an even closer reference in the SNP discovery process [63]. Additional comparative evidence can be obtained from two studies reporting SNP discovery and high-throughput validation in the channel catfish [44] and the Atlantic cod [45]. In both studies, validation was carried out using Illumina GoldenGate technology on either 384 catfish SNPs or 3,072 cod SNPs. Assay conversion rates were similar (266/384 (69.2%) in catfish, 2,291/3,072 (74.5%) in cod) and intermediate between those observed here and values obtained for turkey and mallard. However, SNP discovery was based on EST libraries produced with traditional Sanger sequencing (with higher read quality), therefore the results are not entirely comparable.

Of the successfully converting assays, a slightly (non-significantly) higher percentage (67.5% vs 63.3%, χ^2^ = 1.18, p = 0.272) of truly polymorphic sites was detected in GAII-SNPs (Table 3). These values are similar to those described in the channel catfish (156/266, 58.6%) [44] and the Atlantic cod (1,684/2,291, 73.5%) [45], although yet again these two studies are not entirely comparable. For instance, size and composition of the discovery panel for cod and catfish could not be determined. When comparing rates of hake polymorphic SNPs with other NGS-based SNP discovery studies, the percentage obtained from the transcriptome of sockeye salmon (11/51, 21.5%) [41] is lower, but those reported for RRL sequencing in the turkey (324/340, 95.2%) and the mallard (363/364, 99.7%) are significantly higher. Size and composition of the discovery panel appear similar (10 individuals from 5 populations in the sockeye salmon, 6 unrelated individuals in the turkey, 9 individuals from 3 populations in the mallard, 5–8 individuals from 4 locations in the hake), while the choice between transcriptome and genome sequencing as well as the availability and quality of reference sequences vary across studies. As already observed for the assay conversion rate, the relevance of the latter factor is emphasized by the significant difference (χ^2^ = 5.62, p = 0.017) in the percentage of polymorphic “unmapped” SNPs (20/24, 83.3%) and “mapped” ones (304/316, 96.3%) in the turkey data set [62]. Flanking sequences (120 bp) of SNPs validated as polymorphic in this study are available in Table S3.

**Figure 4:**
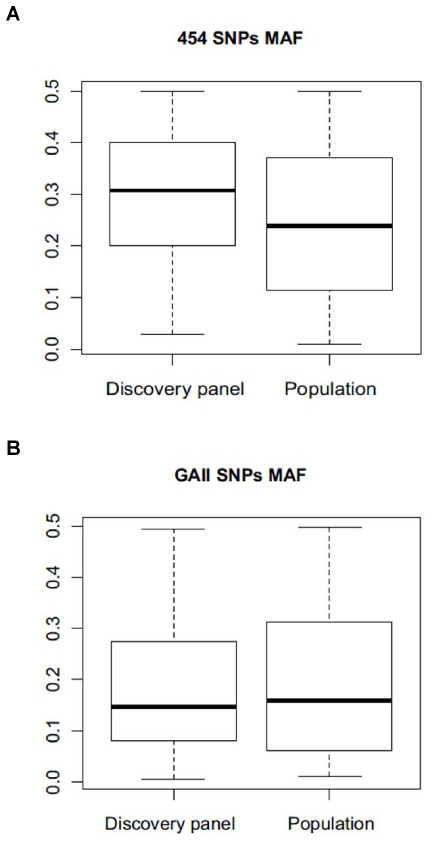
Minor allele frequency distribution. Box plot of minor sequence allele frequency (MSAF) in the discovery panel and Minor allele frequency (MAF) in the validation panel for 454 data (A) and GAII data (B).

**Figure 5:**
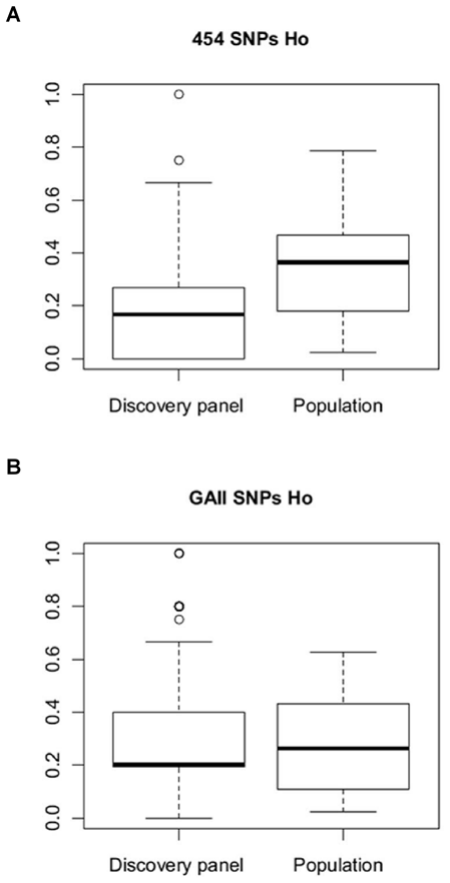
Observed heterozygosity distribution. Box plot of observed heterozygosity (Ho) calculated for the discovery panel and the validation panel of 454 data (A) and GAII data (B).

While similar conversion rates and percentages of polymorphic loci were obtained for both NGS technologies applied to hake SNP discovery, 454-SNPs showed a significantly higher number of Blast matches against at least one fish model coding sequence (*I_E_test* rate 38.4% (454) – 17.9% (GAII), χ^2^ = 68.05 p<0.0001). Also continuous/ordinal variables were significantly different between the two data sets (data not shown), with average *I_E_species_match* and *MSAF* being higher in 454 SNPs, whereas mean *SNP_score*, *Geosites*, and *Depth* were larger in GAII data. Higher mean number of species matching with 454-SNPs is linked to the significantly larger *I_E test* rate, which in turn likely correlates with the higher percentage of annotated 454-contigs, while greater *Depth* in GAII-SNPs reflects the overall much deeper sequence coverage obtained with GAII technology. Likewise, differential coverage likely explains the more complete representation of sequence variation across all four collection sites in GAII-SNPs. It is not clear why higher average *SNP_score* for GAII-SNPs was observed. It might be an effect of different contig length and/or sequence quality as the parameters that the ADT uses for estimating SNP scores are likely influenced by these factors. The higher *MSAF* estimated for 454-SNPs appears to be the effect of over-estimation of allele frequencies based on sequence data, again putatively related to the lower coverage of SNP sites obtained with 454 technology. When sequence-based and Illumina Golden Gate genotypic results are compared for the same loci in a paired-samples Wilcoxon signed rank test, Minor Allele Frequency (*MAF*) is significantly lower than *MSAF* (Wilcoxon paired rank test V = 22,047.5, p<0.0001) (Figure 4A), whereas it is not different for GAII-SNPs (Wilcoxon paired rank test V = 9,503, p = 0.50) (Figure 4B). Neither method provides accurate values of observed heterozygosity (Ho), although GAII-based data tend to over-estimate Ho (Wilcoxon paired rank test V = 12,830, p<0.001, Figure 5B), whereas 454-based Ho provides a substantial under-estimate of this parameter (Wilcoxon paired rank test V = 7,372, p<0.00001, Figure 5A).

To evaluate the predictive value and thus the potential usefulness of different parameters, all variables were examined together using binomial logistic regression analysis. The success rate for Illumina genotyping assay conversion/clustering show that inclusion of all predictor variables significantly improves model-fitting for 454 (χ^2^ = 34.944, degrees of freedom (df) 9, p<0.001) and marginally for GAII data (χ^2^ = 14.641, df 7, p<0.05). The ability to predict the outcome of individual SNP assays is relatively low, with an overall correct classification rate of 60.1% for 454 data and 56.7% for GAII data. Backward stepwise deletion of predictor variables identifies five best predictors (*SNP_score*, *I_E_test*, *MSAF*, *Individuals*, *Q*) for 454 data and three predictors (*SNP_score*, *Individuals*, *Geosites*) for GAII data as summarized in Tables 4 and 5. The number of individuals effectively sequenced for each SNP is positively correlated with the rate of successfully converting assays in both data sets. This is especially significant for GAII data (Table 5), where the maximum number of animals in the discovery panel (5) is lower than in the 454 one (8), suggesting that sequencing coverage *per se* is less relevant than the level of coverage across different individuals. The outcome of the intron-exon boundary pipeline is significant as a predictor only for 454 data, with a higher number of successful assays for SNPs that are verified to be located in a single exon compared to “unknown” SNPs. This is in agreement with what reported by Wang and et al. [44] in the channel catfish, where the presence of an intron was among the causes of assay failure. Comparative genomic analysis to preliminarily exclude SNP candidate regions that are putatively interrupted by one or more introns might therefore be advisable, although it appears not significant for GAII-SNPS, likely in consequence of the lower number of positive Blast matches (Table 2). Less clear is the interpretation for the positive correlation of conversion rate with *MSAF* in 454-SNPs and the negative correlation with number of geographic areas in GAII-SNPs, while the negative correlation of the variable Q, which reports the number of mismatches in the flanking regions of SNP positions based on comparison of sequence reads within a contig, can be easily explained as a result of poor sequence quality on assay performance. This parameter was estimated only on 454-SNPs because the much greater sequencing depth of GAII data did not allow visual estimation of Q. Since this variable appears to convey valuable information, it might be useful to develop an automated pipeline to measure it in the future. Finally, for both data sets, and in particular for 454-SNPs, the ADT score proved to be a significant predictor (Tables 4 and 5).

**Table 4:** Predictor variables for 454 SNP data (failed/successful), backward stepwise elimination.

	B^a^	Wald^b^	df	P^c^
**SNP_score**	1.735	14.607	1	**0.000**
**I_E_test(1)**	−0.361	6.545	1	**0.011**
**MSAF**	1.415	5.418	1	**0.020**
**Individuals**	0.075	2.919	1	0.088
**Q**	−0.007	5.205	1	**0.023**
**Constant**	−2.339	13.067	1	0.000

a Regression coefficient for individual variable,

b Wald χ^2^ statistic,

c associated probability. (*e.g.* average SNP_score for successful SNPs is 0.794, whereas mean SNP_score is 0.759 for “failed” assays).

**Table 5:** Predictor variables for GAII SNP data (failed/successful), backward stepwise elimination.

	B^a^	Wald^b^	df	P^c^
**SNP_score**	0.938	2.506	1	0.113
**Individuals**	1.203	8.795	1	**0.003**
**Geosites**	−1.206	7.427	1	**0.004**
**Constant**	−2.196	6.785	1	0.009

a Regression coefficient for individual variable,

b Wald χ^2^ statistic,

c associated probability.

A specific statistical analysis (Receiver Operating Characteristic (ROC) analysis) was carried out to evaluate the significance of ADT score as predictive variable. The estimated area under the ROC curve is 0.539±0.015, which is significantly different (z statistic = 2.6, p<0.01) than expected by chance (0.5), confirming ADT score as a significant predictor of assay conversion. The optimal ADT value, which has the best specificity/sensitivity ratio, is 0.735. It should be noted that, while significant, the overall performance of ADT as diagnostic factor is rather limited.

**Figure 6:**
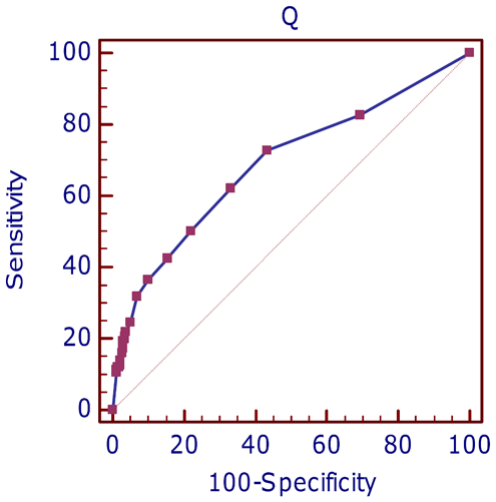
ROC curve of Q score predicting monomorphic/polymorphic SNPs in 454 data.

Binomial logistic regression analysis was also implemented to evaluate the outcome of successful SNP assays, *i.e.* to predict polymorphic and monomorphic loci. Including all predictor variables, model fitting improved significantly for 454 data (χ^2^ 55.983, df 9, p<0.0001) as well as for GAII data (χ^2^ 29.059, df 8, p<0.001), with overall rates of correct classification of 70.9% (454) and 70.6% (GAII). Stepwise deletion of independent variables reduced the list of contributing predictors to *I_E_species_match*, *Rank*, *Individuals*, *Geosites*, and *Q* in the case of 454 data, while *I_E_test*, *I_E_species_match*, *Individuals*, and *Geosites* best predicted *SNP_genotype* for GAII data (Tables 6 and 7). The three predictive variables that are common to the two data sets show opposite correlations with SNP polymorphism, which suggests caution in using them as predictors, while the negative correlation of *I_E_test* outcome with polymorphism in GAII-SNPs is not easily interpreted and is of little use as positive *I_E_test* is relevant for predicting assay conversion, at least in 454-SNPs. More interesting is the negative correlation between the number of mismatches in the flanking regions (*Q*) with the degree of polymorphism, similar to what was observed for assay conversion rate. This evidence is in agreement with the positive predictive value of *Rank*, a subjective measure of overall candidate SNP suitability, which also takes into account contig sequence quality. While *Rank* cannot be translated into an objective, operator-free score, *Q* appears a promising predictor, which might be possible to automatically estimate for a very large number of SNPs and could provide useful information on assay conversion as well as SNP polymorphism. In fact, ROC analysis shows that the area under the ROC curve (0.688±0.0279, Figure 6, see below) is highly significant for *Q* when predicting whether a specific locus is monomorphic (z statistic = 6.737, p<0.0001). Optimal trade-off between specificity (56.7%) and sensitivity (72.7%) is obtained with *Q*>1.

**Table 6:** Predictor variables for 454 SNP data (monomorphic/polymorphic), backward stepwise elimination.

	B^a^	Wald^b^	df	P^c^
**I_E_speciesmatch**	−0.127	6.369	1	**0.012**
**Rank**		6.977	2	**0.031**
**Rank(1)**	0.676	4.232	1	**0.040**
**Rank(2)**	0.841	4.983	1	**0.026**
**Individuals**	−0.224	3.489	1	0.062
**Geosites**	0.802	10.433	1	**0.001**
**Q**	−0.044	8.907	1	**0.003**
**Constant**	−2.740	5.915	1	0.015

a Regression coefficient for individual variable,

b Wald χ^2^ statistic,

c associated probability.

**Table 7:** Predictor variables for GAII SNP data (monomorphic/polymorphic), backward stepwise elimination.

	B^a^	Wald^b^	df	P^c^
**I_E_test(1)**	2.247	8.272	1	**0.004**
**I_E_speciesmatch**	0.345	2.735	1	0.098
**Individuals**	1.805	5.162	1	**0.023**
**Geosites**	−2.188	5.581	1	**0.018**
**Constant**	−1.577	1.361	1	0.243

a Regression coefficient for individual variable,

b Wald χ^2^ statistic,

c associated probability.

Finally, all experimentally validated SNPs were analysed using a bespoke pipeline, which was developed to compare SNP-containing contigs with known protein sequences and to predict protein-coding regions and the corresponding putative reading frame. It was possible to obtain this information reliably for approximately half of the validated SNPs (Table 3) for both data sets, a much higher percentage than that obtained for Atlantic cod (9%) [45]. This is likely attributable to the less 5′ end-biased transcript coverage of NGS technologies compared to Sanger ESTs. Of all SNPs located in a coding region, around 20% were putative amino acid replacement substitutions, without significant difference between 454- and GAII-SNPs (Table 3). In this respect, Atlantic cod SNPs were significantly biased toward non-synonymous substitutions (synonymous/non-synonymous 90/51) compared to all hake SNPs (147/28, after excluding common loci) (χ^2^ 15.06, df 1, p<0.0001). Closer examination of non-synonymous SNPs shows that, if amino acids are divided into five classes (non-polar, polar, negatively-charged, positively-charged, and aromatic), the majority of amino acid substitutions are conservative replacements (*i.e.* occurring within the same class), but there are 12 non-conservative mutations, which might cause significant functional changes in the encoded protein.

### Conclusions

In the present study, two different NGS methods were applied to high-throughput SNP discovery in the muscle transcriptome of a non-model fish species. Overall, the comparison revealed that despite substantial differences in sequence throughput, average sequencing depth, and sequence read length, similar results were obtained after SNP experimental validation in terms of assay conversion rate and percentage of polymorphic loci. GAII technology yields a larger number of candidate SNPs, but the majority of them are not suitable for SNP genotyping due to short flanking regions. On the other hand, 454-SNPs are less numerous, but are located on longer contigs, which are more easily annotated and screened for putative introns in the candidate region using a comparative genomic approach. Although the platforms we have evaluated have been recently upgraded and superseded by later versions, still our findings should remain valid and relevant. Indeed, during the past few years Illumina and 454 platforms have experienced rapid progress towards the enhancement of reads length and yield in terms of Mb per run produced, mainly resulting in increased throughput; however, raw sequencing error rates have not decreased along with the improvement of instruments and chemistry [64]. For this reason, while using latest platforms could increase transcriptome representation and coverage and improve *de novo* assembly, due to the higher sequencing output, we believe results on SNP discovery and validation, primarily influenced from sequencing error profiles, would be proportionately comparable to our results. In order to evaluate and compare costs of SNP discovery, several aspects should be taken into account. However, considering only the raw cost of reagents per Mb updated to 2011 [64], and referring to the output produced and used in this study for SNP discovery, the Illumina sequencing systems seems to be more appropriate, as similar results in terms of validated SNPs were obtained at less than half the 454 costs (480$ to produce approximately 4,000 Mbp at a reference price of 0.12$/Mbp using the Illumina GAIIx against 1240$ to produce approximately 100 Mbp at a reference price of 12.4$/Mbp using the 454 FLX Titanium; based on [64]). If we consider the respective platforms currently available, the Illumina HiSeq 2000-v3 and the 454 FLX+, the cost-effectiveness of the Illumina technology is maintained, as same results would be achieved with less than a quarter of 454 costs. Additionally, HiSeq technology might allow to increase the number of individuals included in the SNP discovery panel without decreasing coverage depth. As such a variable (Individuals) was found to be positively correlated with successful conversion rate of SNP assays, this represents a further element in favor of Illumina technology. It should be noted, however, that the most expensive steps were the *in silico* analysis of sequence reads (assembly, SNP discovery, quality assessment) and the high-throughput genotyping assays. While the latter showed similar conversion rates, analysis of GAII reads might be more laborious and produce less reliable assemblies [47]. On the other hand, it has been demonstrated that the quality of transcriptome assembly depends on sequence coverage [47], therefore the shift toward HiSeq might allow easier and more reliable assemblies. More generally, the results presented here clearly demonstrate that it is possible to identify and effectively validate many polymorphic SNPs, in transcribed regions of a non model species. The lack of a reference genome, however, dramatically affects the genotyping success rate, although the overall efficiency can be improved using strict quality criteria/filters, especially in the case of 454-SNPs, such as testing for intron-exon boundaries, defining optimal ADT scores, and targeting low *Q* values (number of mismatches in the flanking regions).

The SNP markers developed in the current study represent novel tools for a broad range of future applications in population genetic studies focusing on the European hake. A deeper understanding of ecological and evolutionary dynamics of European hake populations across the entire distribution range provides the necessary means for a proper management and conservation policy, aimed at promoting sustainable fishery and preventing overexploitation and illegal fishing activities.

## Supporting Information

Figure S1 **Gene Ontology (GO) assignment (2nd level GO terms) of hake 454 contigs.** **A** “Cellular Component”. **B** Molecular Function”. **C** “Biological Process”.
(PDF)

Figure S2 **Gene Ontology (GO) assignment (2nd level GO terms) of hake GAII contigs.** **A** “Cellular Component”. **B** Molecular Function”. **C** “Biological Process”.
(PDF)

Figure S3 **Flowchart describing the intron-exon pipeline.**
(PDF)

Table S1 **Results of GO enrichment analysis performed using all 454 contigs as reference set, and 454 SNP-containing contigs as test set.** Significantly overrepresented GO terms are listed, together with the respective category (P: “Biological Process”; F: “Molecular Function”; C: “Cellular Component”), FDR (false discovery rate) and Fisher's Exact Test p-value.
(PDF)

Table S2 **Results of GO enrichment analysis performed using all GAII contigs as reference set, and GAII SNP-containing contigs as test set.** Significantly overrepresented GO terms are listed, together with the respective category (P: “Biological Process”; F: “Molecular Function”; C: “Cellular Component”), FDR (false discovery rate) and Fisher's Exact Test p-value.
(PDF)

Table S3 **List of 395 SNPs validated as polymorphic in this study (including 454-, GAII- SNPs and the set of loci detected using both data sets) together with the corresponding flanking sequences of approximately 120 bp (SNP alleles in brackets).**
(XLSX)
