# Supplementary figures and images for: Novel Tools for Conservation Genomics: Comparing Two High-Throughput Approaches for SNP Discovery in the Transcriptome of the European Hake
Source: PLoS One. 2011 Nov 22;6(11):e28008. doi: 10.1371/journal.pone.0028008 (PMC3222667; doi:10.1371/journal.pone.0028008)

**A**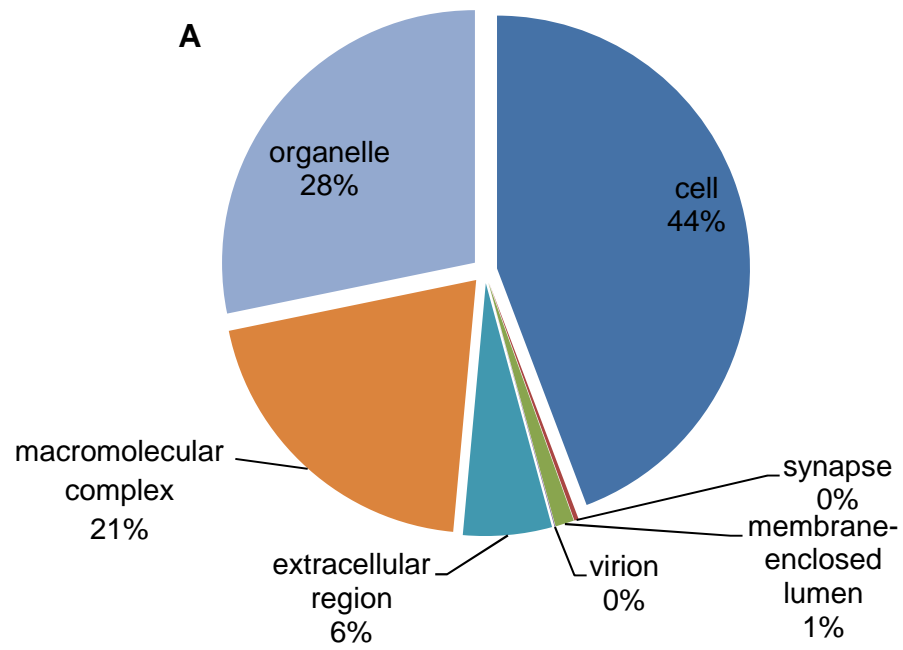**B**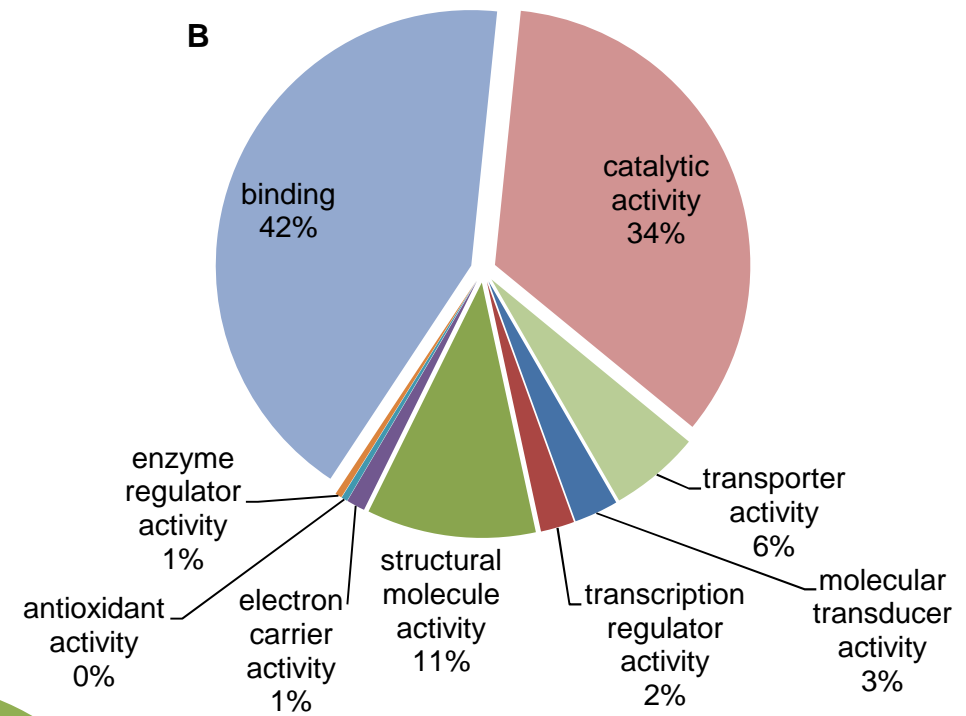**C**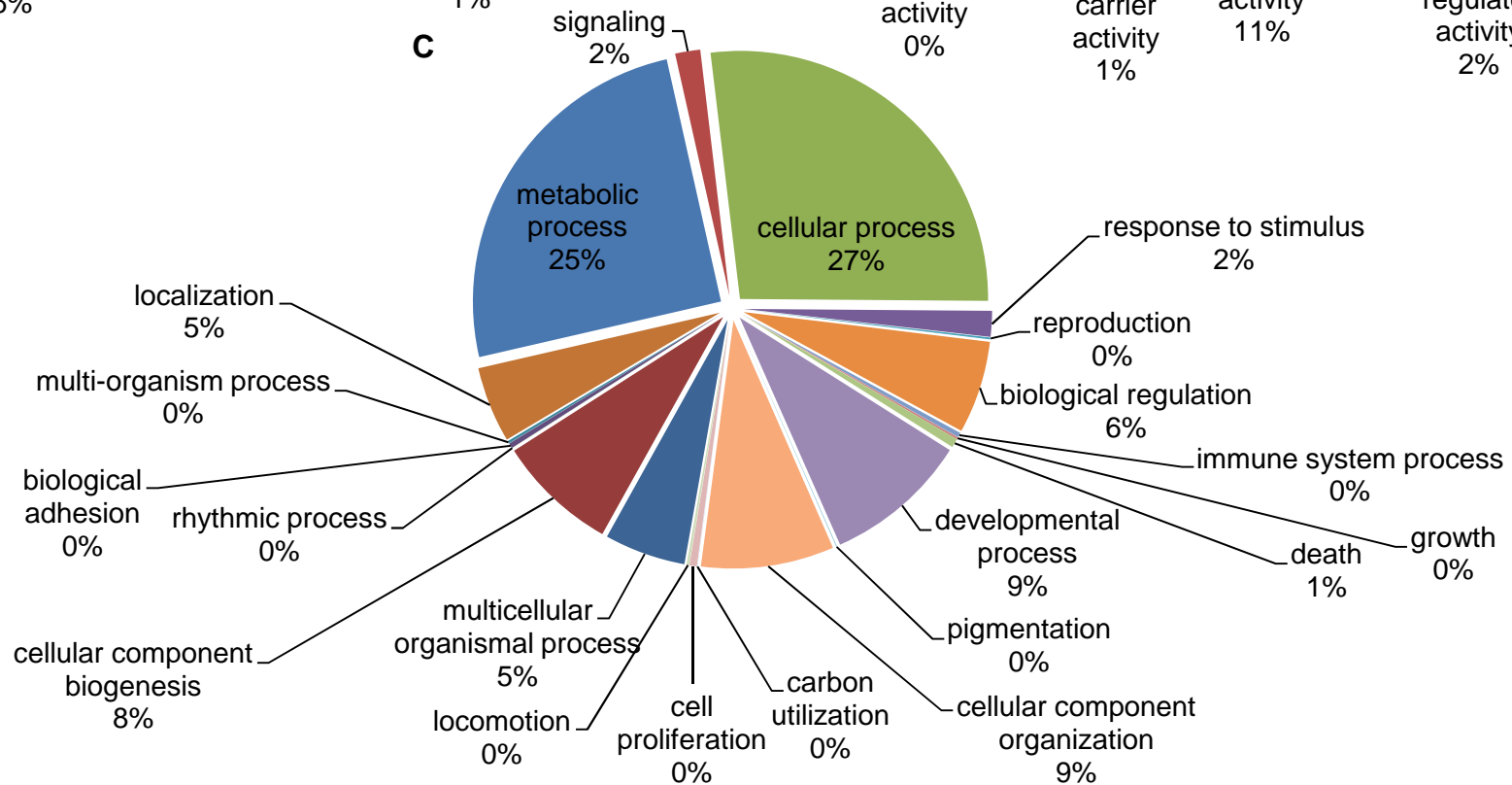

Supplement: Figure S1 — Gene Ontology (GO) assignment (2nd level GO terms) of hake 454 contigs. A “Cellular Component”. B Molecular Function”. C “Biological Process”. (PDF) [file pone.0028008.s001.pdf]

**A**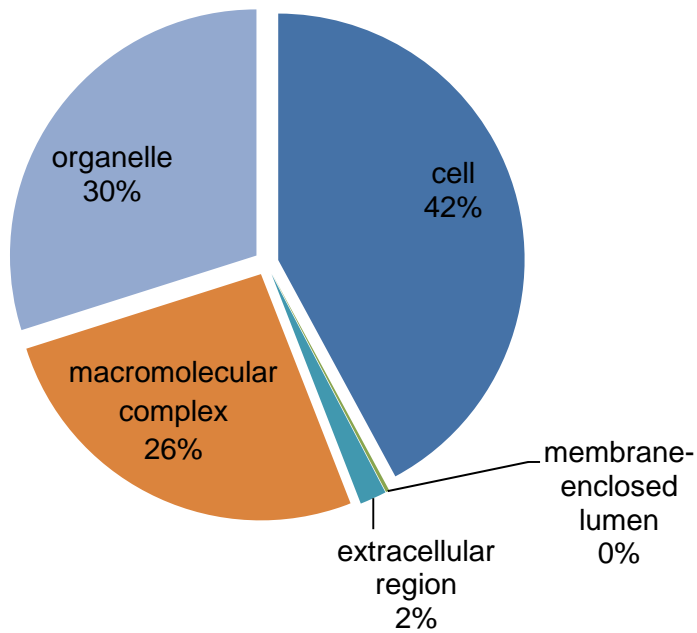**B**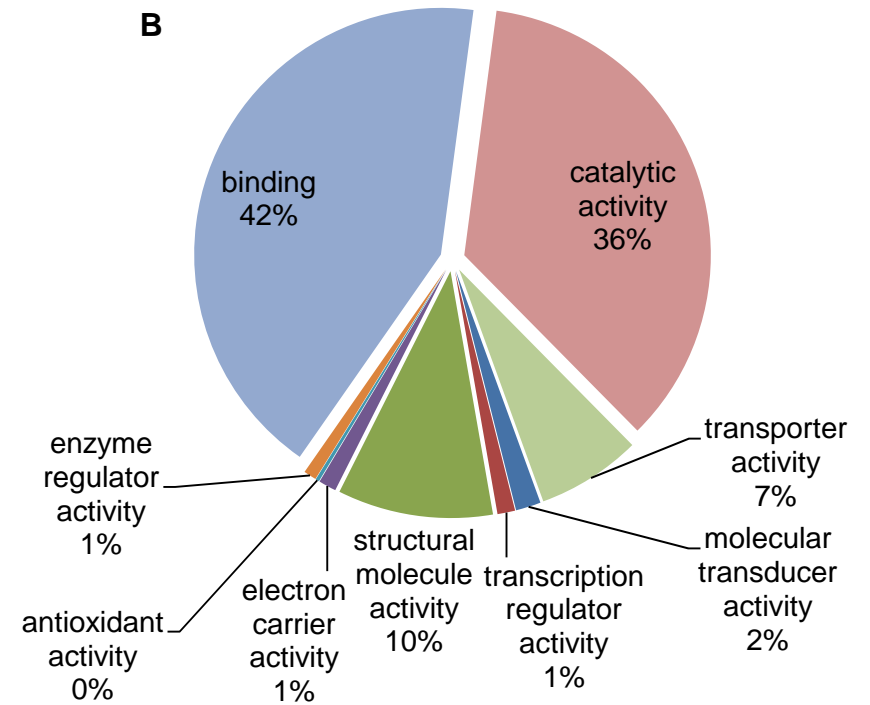**C**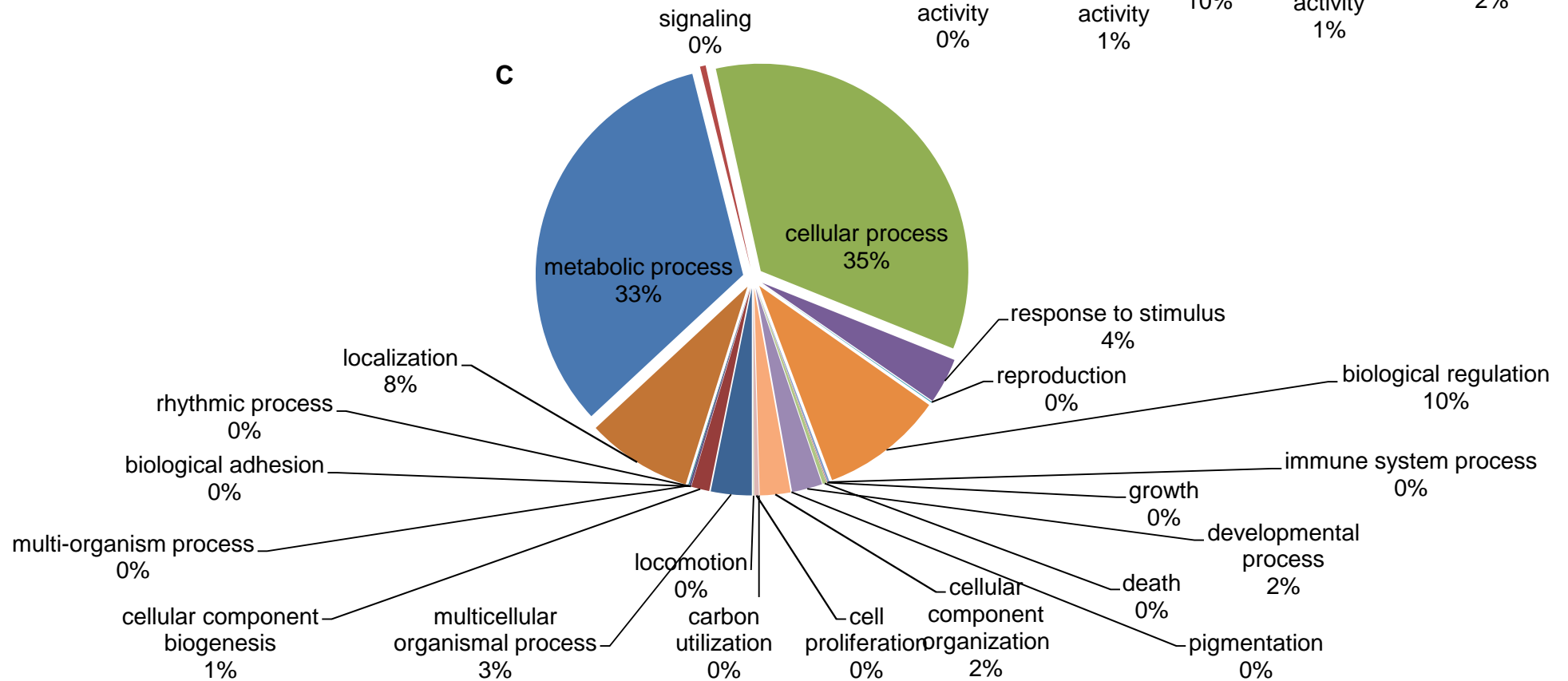

Supplement: Figure S2 — Gene Ontology (GO) assignment (2nd level GO terms) of hake GAII contigs. A “Cellular Component”. B Molecular Function”. C “Biological Process”. (PDF) [file pone.0028008.s002.pdf]

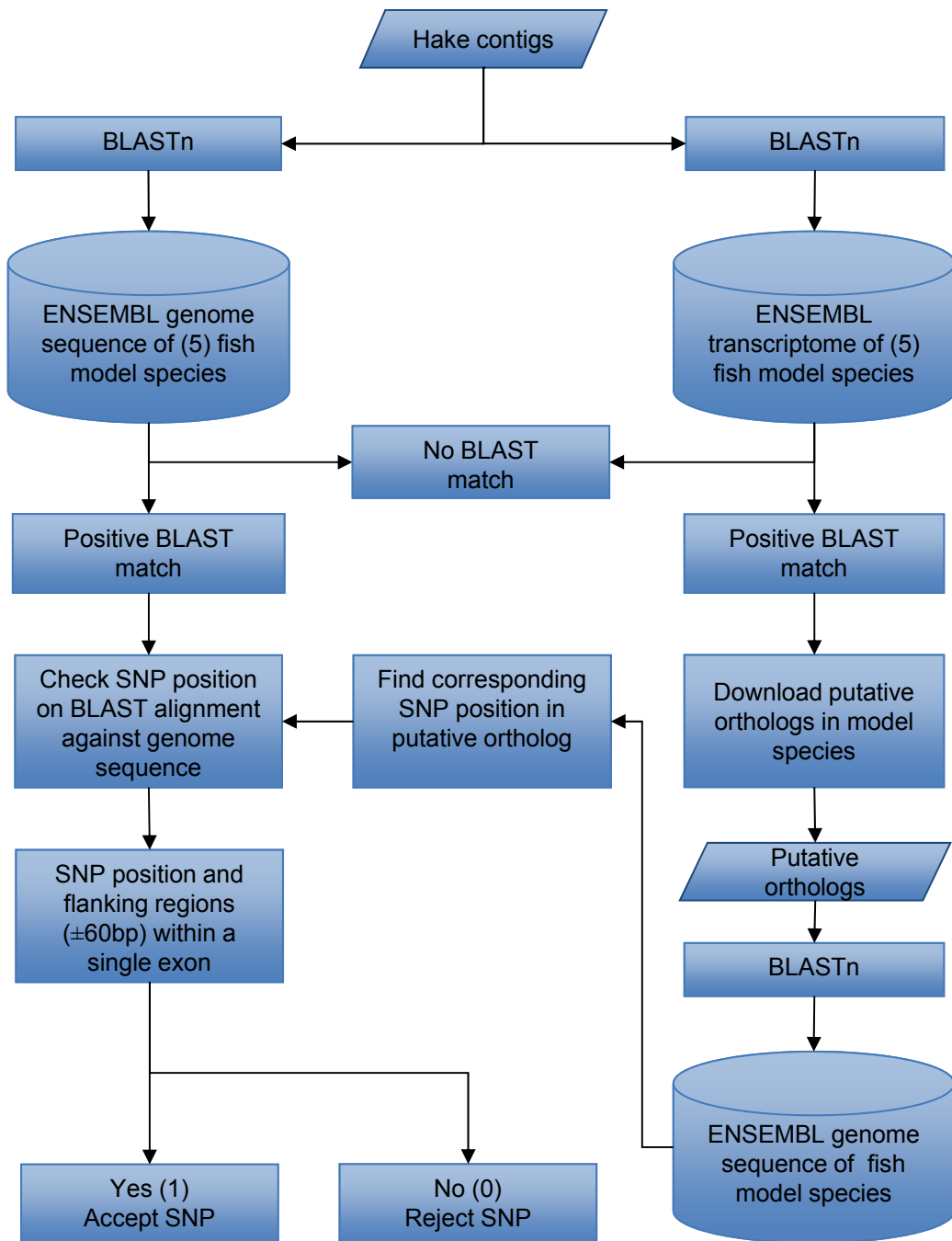

Supplement: Figure S3 — Flowchart describing the intron-exon pipeline. (PDF) [file pone.0028008.s003.pdf]
