# Supplementary material for: Novel Tools for Conservation Genomics: Comparing Two High-Throughput Approaches for SNP Discovery in the Transcriptome of the European Hake
Source: PLoS One. 2011 Nov 22;6(11):e28008. doi: 10.1371/journal.pone.0028008 (PMC3222667; doi:10.1371/journal.pone.0028008)
Supplement: Table S1 — Results of GO enrichment analysis performed using all 454 contigs as reference set, and 454 SNP-containing contigs as test set. Significantly overrepresented GO terms are listed, together with the respective category (P: “Biological Process”; F: “Molecular Function”; C: “Cellular Component”), FDR (false discovery rate) and Fisher's Exact Test p-value. (PDF) [file pone.0028008.s004.pdf]

GO-ID	Term	Category	FDR	P-Value	Over/Under
GO:0006412	translation	P	2.40E-04	0.0	over
GO:0003735	structural constituent of ribosome	F	2.40E-04	0.0	over
GO:0005840	ribosome	C	2.40E-04	0.0	over
GO:0030529	ribonucleoprotein complex	C	2.40E-04	1.32E-08	over
GO:0009058	biosynthetic process	P	2.40E-04	6.80E-08	over
GO:0034645	cellular macromolecule biosynthetic process	P	2.40E-04	9.78E-08	over
GO:0009059	macromolecule biosynthetic process	P	2.40E-04	9.78E-08	over
GO:0044249	cellular biosynthetic process	P	2.40E-04	1.88E-07	over
GO:0044444	cytoplasmic part	C	5.72E-03	2.62E-04	over
GO:0043232	intracellular non-membrane-bounded organelle	C	1.00E-01	4.90E-03	over
GO:0043228	non-membrane-bounded organelle	C	1.00E-01	4.90E-03	over
GO:0005198	structural molecule activity	F	7.90E+00	3.91E-01	over
GO:0032991	macromolecular complex	C	0.00200569	8.51E+00	over
GO:0033279	ribosomal subunit	C	0.0113124	5.11E+01	over
GO:0006091	generation of precursor metabolites and energy	P	0.0113124	5.19E+01	over
GO:0016868	intramolecular transferase activity, phosphotransferases	F	0.02043	8.30E+01	over
GO:0044424	intracellular part	C	0.0231671	9.97E+01	over
GO:0005975	carbohydrate metabolic process	P	0.0332324	0.00171534	over
GO:0022900	electron transport chain	P	0.0332324	0.00178515	over
GO:0016866	intramolecular transferase activity	F	0.0344973	0.00197345	over
GO:0006414	translational elongation	P	0.0350837	0.00206099	over
GO:0030170	pyridoxal phosphate binding	F	0.0422907	0.00290272	over
GO:0022904	respiratory electron transport chain	P	0.0422907	0.00290272	over
GO:0070279	vitamin B6 binding	F	0.0422907	0.00290272	over
GO:0008152	metabolic process	P	0.0422907	0.00292836	over
