# Supplementary material for: Novel Tools for Conservation Genomics: Comparing Two High-Throughput Approaches for SNP Discovery in the Transcriptome of the European Hake
Source: PLoS One. 2011 Nov 22;6(11):e28008. doi: 10.1371/journal.pone.0028008 (PMC3222667; doi:10.1371/journal.pone.0028008)
Supplement: Table S2 — Results of GO enrichment analysis performed using all GAII contigs as reference set, and GAII SNP-containing contigs as test set. Significantly overrepresented GO terms are listed, together with the respective category (P: “Biological Process”; F: “Molecular Function”; C: “Cellular Component”), FDR (false discovery rate) and Fisher's Exact Test p-value. (PDF) [file pone.0028008.s005.pdf]

GO-ID	Term	Category	FDR	P-Value	Over/Under
GO:0003735	structural constituent of ribosome	F	5.73E-01	9.95E-03	over
GO:0030529	ribonucleoprotein complex	C	5.73E-01	1.59E-02	over
GO:0032991	macromolecular complex	C	5.73E-01	1.67E-02	over
GO:0005840	ribosome	C	8.87E-01	4.06E-02	over
GO:0043228	non-membrane-bounded organelle	C	8.87E-01	5.62E-02	over
GO:0043232	intracellular non-membrane-bounded organelle	C	8.87E-01	5.62E-02	over
GO:0005198	structural molecule activity	F	2.57E+00	1.71E-01	over
GO:0016459	myosin complex	C	9.29E+00	6.18E-01	over
GO:0006412	translation	P	1.38E+00	1.11E+00	over
GO:0034645	cellular macromolecule biosynthetic process	P	1.38E+00	1.28E+00	over
GO:0009059	macromolecule biosynthetic process	P	1.38E+00	1.28E+00	over
GO:0003779	actin binding	F	1.41E+01	1.55E+00	over
GO:0008092	cytoskeletal protein binding	F	1.41E+01	1.55E+00	over
GO:0003774	motor activity	F	4.85E+01	4.79E-01	over
GO:0032982	myosin filament	C	5.64E+01	6.45E+00	over
GO:0044430	cytoskeletal part	C	8.65E+01	9.14E+00	over
GO:0044424	intracellular part	C	8.98E+01	1.03E+01	over
GO:0044422	organelle part	C	0.00153193	2.07E+01	over
GO:0044446	intracellular organelle part	C	0.00153193	2.07E+01	over
GO:0016818	hydrolase activity, in phosphorus-containing anhydrides	F	0.00512314	6.35E+01	over
GO:0017111	nucleoside-triphosphatase activity	F	0.00512314	6.35E+01	over
GO:0016462	pyrophosphatase activity	F	0.00512314	6.35E+01	over
GO:0016817	hydrolase activity, acting on acid anhydrides	F	0.00644593	8.50E+01	over
GO:0005515	protein binding	F	0.00721798	0.00109514	over
GO:0044464	cell part	C	0.0107931	0.00153039	over
GO:0044249	cellular biosynthetic process	P	0.0139449	0.00177106	over
GO:0009058	biosynthetic process	P	0.0222579	0.00294704	over
GO:0005524	ATP binding	F	0.0401276	0.00505448	over
GO:0032559	adenyl ribonucleotide binding	F	0.0401276	0.00505448	over
GO:0032555	purine ribonucleotide binding	F	0.0434069	0.00597614	over
GO:0032553	ribonucleotide binding	F	0.0434069	0.00597614	over
GO:0016787	hydrolase activity	F	0.047443	0.00745794	over
GO:0043226	organelle	C	0.047443	0.00751754	over
GO:0043229	intracellular organelle	C	0.047443	0.00751754	over
GO:0030554	adenyl nucleotide binding	F	0.047443	0.0080511	over
GO:0001883	purine nucleoside binding	F	0.047443	0.0080511	over
GO:0001882	nucleoside binding	F	0.047443	0.0080511	over
